# Detection and identification of *Legionella* species in hospital water supplies through Polymerase Chain Reaction (16S rRNA)

**DOI:** 10.1186/2052-336X-12-83

**Published:** 2014-05-09

**Authors:** Mohammad Rafiee, Mahsa Jahangiri-rad, Homa Hajjaran, Alireza Mesdaghinia, Mohammad Hajaghazadeh

**Affiliations:** 1 Department of Environmental Health Engineering, School of Public Health, Alborz University of Medical Sciences, Alborz, Iran; 2 Department of Environmental Health Engineering, Islamic Azad University, Tehran Medical Sciences Branch, Tehran, Iran; 3 Department of Parasitology and Mycology, School of Public Health, Tehran University of Medical Sciences, Tehran, Iran; 4 Department of Environmental Health Engineering, School of Public Health, Center for Water Quality Research, Institute for Environmental Research, Tehran University of Medical Sciences, Tehran, Iran; 5 Department of Occupational Health, Health Faculty, Urmia University of Medical Sciences, Urmia, Iran

**Keywords:** *Legionella*, Hospital water supplies, DNA extraction, PCR, 16S rRNA

## Abstract

*Legionella* spp. are important waterborne pathogens that are normally transmitted through aerosols. The present work was conducted to investigate the presence of *Legionella* spp. and its common species in hospital water supplies. Considering the limitations of culture method, polymerase chain reaction (PCR) assays were developed to detect the gene 16S rRNA irrespective of the bacterial serotype. Four well-established DNA extraction protocols (freeze & thaw and phenol-chloroform as two manual protocols and two commercial kits) were tested and evaluated to release DNA from bacterial cells. A total of 45 samples were collected from seven distinct hospitals’ sites during a period of 10 months. The PCR assay was used to amplify a 654-bp fragment of the 16S rRNA gene. *Legionella* were detected in 13 samples (28.9%) by all of the methods applied for DNA extraction. Significant differences were noted in the yield of extracted nucleic acids. *Legionella* were not detected in any of the samples when DNA extraction by freeze & thaw was used. Excluding this method and comparing manual protocol with commercial kits, Kappa coefficient was calculated as 0.619 with *p* < 0.05. Although no meaningful differences were found between the kits, DNA extraction with Bioneer kit exhibited a higher sensitivity than classical Qiagen. Showerheads and cold-water taps were the most and least contaminated sources with 55.5 and 9 percent positive samples, respectively. Moreover two positive samples were identified for species by DNA sequencing and submitted to the Gene Bank database with accession Nos. FJ480932 and FJ480933. The results obtained showed that despite the advantages of molecular assays in *Legionella* tracing in environmental sources, the use of optimised DNA extraction methods is critical.

## Introduction

*Legionella* are thin, gram-negative, obligate aerobic and sporeless rods with complex nutritional requirements. Certain species of *Legionella* like *Legionella pneumophila* are often strongly associated with asymptomatic infections (Legionnaires' disease) or produce mild cough, sore throat and fever (Pontiac fever) that goes away by itself in a 2-5 day period. The term "legionellosis" may be used to refer to either Legionnaires' disease or Pontiac fever. However, more than 10 known serotypes are implicated in severe pulmonary nosocomial infections, especially in immunocompromised patients as well as in the elderly and subjects already suffering from pulmonary diseases [1-3]. Indeed, twenty-one species of *Legionella* are pathogens for humans, especially in patients with chronic pulmonary disease within hospitals [4]. The bacterium can be isolated from aquatic and terrestrial habitats as well as from legionellosis patients [3]. Disease occurs after exposure to aquatic settings that promote bacterial growth-the aquatic environment is somewhat stagnant, the water is warm (25°C–42°C), and the water must be aerosolized so that the bacteria can be inhaled into the lungs. Inhalation or micro aspiration of *Legionella* from contaminated environmental sources such as hot water systems and cooling towers’ water is the most frequent route of transmission. While transmission has also been reported via nebulizer and showers in contaminated water, the infection is not spread from person to person [3,5,6].

Outbreaks of legionellosis have been described in numerous countries throughout the world. In 2007, there were 2716 reported cases, near 8 cases per million in the United States (CDC) [7]. Travel-associated outbreaks are commonly recognized [8]. CDC estimates that between 8,000 and 18,000 people are hospitalized with LD in the United States each year [9]. Hospitals are common habitats for the bacterium, where the bacterial niches are amply found and provide the most likely places for susceptible people to contract the diseases. Outbreaks of legionellosis have been reported from hospital patients in many countries with an incidence range of 0 to 47% [10,11]. Consequently, national *Legionella* surveillance programs have been established for regular monitoring of environmental samples in these countries [12,13]. In Iran however, hospital-acquired Legionnaire's Disease has rarely been reported and environmental surveillance for *Legionella* in hospital water systems to provide useful data for risk assessment and prevention has never been systematically performed.

The polymerase chain reaction (PCR) is considered the most adaptable and prevalent DNA-based assay technique, which is a highly specific and sensitive alternative method to standardize culture isolation, especially when rapid results are required. This method is especially favorable when the samples contain abundant and diverse micro biota and when fastidious and slow-growing bacteria like *Legionella* need to be detected. So despite the fact that culture method for isolation of *Legionella* is the golden standard and has been approved by ISO and many other national standards for water quality determination, over the past few years, molecular techniques based on 16S rRNA gene besides other genetic markers have been developed to analyze bacterial communities in environmental samples [14,15]. Indeed, considering the complexity of bacterial behaviors which are not easily predictable, PCR can be economically profitable but requires attention in evaluating the suitability and consistency of the used tools and recipes in order to select the best appropriate and efficient ones and consequently, achieving the best results. It has been demonstrated that the application of PCR depends on the extraction of DNA from the organisms, which is often the most critical step to avoid false negative PCR results. In general, the extraction of non-degraded and inhibitor-free DNA, suitable for PCR amplification, has been reported as a common issue [16-18]. Several methods including both commercial kits and manual classic protocols have been used for the preparation of *Legionella* DNA from environmental samples and evaluation of the quantity and quality of extracted DNA. Several studies have reported that DNA extraction methods can influence the sensitivity of PCR assays [19-22]. Therefore, selection of the best method for a given sample is of importance for the laboratory detection of given organism. This cross-sectional study was conducted from the above perspective to investigate the presence of bacteria belonging to the *Legionella* genus in water supplies of some hospitals in Tehran, capital city of Iran. The impact of water quality on *Legionella* existence was also determined. In the present study, four well-established but different methods (freeze & thaw, phenol-chloroform, and DNA extraction using two different commercial kits) were tested and evaluated to release DNA from bacterial cells in order to find a reliable method. In spite of the large amount of data available for the various PCR methods to identify *Legionella* species in water samples, to our knowledge, this is the first attempt to gather information on common DNA extraction protocols to monitor the presence of this bacterium and there is no systematic comparative study to assess the relative efficiency of these techniques. For further confirmation, two isolates determined as *Legionella* spp. were randomly sequenced as well.

## Materials and methods

### Sample collection and preparation

A total of forty-five samples were collected from distinct sites at seven teaching hospitals under the auspices of Tehran University of Medical Sciences from June 2011 to January 2012. Hospital water facilities sampled included tap cold and hot water, showerhead, hot water tank, and cooling tower water. Samples were collected in 1-litre sterile bottles directly from the outlet. Before sampling, a sterile swab was inserted into faucet outlets and rotated against the interior surface two times clockwise and up-and-down two times to dislodge the sediment.

### DNA Extraction, PCR assay, Gel electrophoresis, and DNA sequencing

One liter of samples was filtered through 0.22 μm mixed cellulose ester membrane filters (Schleicher & Schuell) in a stainless-steel filter holder with a water aspirator. Each membrane was aseptically scraped, cut into smaller pieces and placed into sterile, screw-capped containers with 10 ml of the original sample. The samples were then sonicated for 5 min (Bandelin Sonorex) at 35 KHz and shaken for 15 min to dislodge bacterial cells from the membranes. The elute was transferred into a 15 mL conical centrifuge tube and centrifuged (2000 g, 20 min) to remove cell debris. Total DNA was extracted from concentrated water samples using two classic manual methods: freeze & thaw and phenol-chloroform as well as two commercial DNA extraction kits: Flexi Gen DNA Kit Qiagen (Hilden, Germany) and Bioneer Accuprep Genomic DNA Kit according to the manufacture's instruction. Table 1 shows the characteristics of these DNA extraction kits. DNA extraction using freeze & thaw method was conducted by placing of 1 mL of each concentrated water sample within 1.5 mL micro tubes and alternating application of freezing the samples in liquid nitrogen and their incubation in water bath in a temperature of 100°C for three or more times. The suspension was then centrifuged again (18000 g, 10 min) and the supernatant was transferred to new micro tubes. Another manual DNA extraction method of phenol-chloroform was done according to Sambrook standard methods [3].

**Table 1:** Characterization of DNA extraction kits

**Company**	**Qiagen**	**Bioneer**
Kit	FlexiGene DNA Kit(50)	Bioneer Accuprep Genomic DNA extraction
Cat. No.	51204	K-3032
No. of preparations	100	100

Extracted DNA was stored at -20°C until using PCR. Amplification reactions were preformed according to what has been described earlier by Hsu [14]. The PCR primers LEG 225 (5'-AAGATTAGCCTGCGTCCGAT-3') and LEG 858 (5'-GTCAACTTATCGCGTTTGCT-3') were used to amplify a 650 bp fragment of the 16S rRNA gene of *Legionella* species. Each 25 μL of reaction contained 20 ng genomic DNA, 1.5 m*M* MgCl_2_ (Roche, Germany), 0.2 m*M* dNTP (Roche, Germany), 20 p*M* of each primer, and 1u of *Tag* DNA polymerase (Roche, Germany) in the PCR buffer (Roche, Germany). The cycling conditions were 94ºC for 5 min, followed by 30 cycles at 95ºC (30 sec), 64ºC and 74ºC for 20 sec each, and 1 cycle of 72ºC for 5 min in Thermocycler (Techne, USA). PCR products were loaded onto a 2% agarose gel containing ethidium bromide. Extracted DNA from cultured *Legionella* was used for positive control. For more confirmation, the PCR products of two *Legionella* isolates were sequenced at MWG (Ebersberg, Germany; http://www.mwg-biotech.com).

### Statistical analysis

Statistical analysis was performed using SPSS (version 9.0; SPSS Inc, Chicago, IL). Quantitative variables were expressed as mean standard deviation when the data was normally distributed, while variables were expressed as median when the data was not in a normal distribution. The results of the DNA extraction tests were reported as qualitative values (positive and negative) and through a comparison of results obtained, the agreement rates were determined by applying the Kappa coefficient.

## Results and discussion

### Comparison among DNA extraction methods

Several studies have compared DNA extraction methods and reported that their abilities to recover bacterial DNA were different, indicating that no single DNA extraction method is optimal for all bacteria [19,20]. In this study, four different procedures (two commercial extraction kits and two classic manual protocols) were evaluated and compared to obtain DNA from hospital water samples. DNA was extracted from water samples using these four methods and followed by PCR amplification of bacterial 16S rRNA gene.

Overall, *Legionella* were detected in 13 samples (28.9%) by all four methods used for DNA extraction. The comparison of these methods regarding their relative ability to extract DNA and detect *Legionella* from samples bore significant differences according to the results obtained (Table 2). As shown in this table, *Legionella* were not detected in any of the samples when DNA extraction by freeze & thaw method was performed. Likewise, samples revealed low levels DNA of *Legionella* (4/45 corresponding to 8.9%) by Qiagen kit. DNA extraction by phenol-chloroform and Bioneer kit, however, revealed the most positive samples for *Legionella*, i.e., 8 out of 45 (17.7%) and 12 out of 45 samples (26.6%) showed contamination with *Legionella*, respectively. Better performance of Bioneer kit may be driven from the fact that columned method was applied for DNA extraction of cells (DNA adsorbed to membrane column), though DNA was precipitated by alcohol in the case of Qiagen kit. Four samples were detected positive by Qiagen DNA extraction kit, which were positive with Bioneer DNA extraction kit too and three of which were also positive with phenol-chloroform. One sample was positive only by phenol-chloroform and four only using Bioneer kit. The 32 resting samples were negative by all three methods.

**Table 2:** ***Legionella *** **prevalence by the source**

**Sampling source**	***Legionella *** **positivity**				
	**(No. of positive/total No.)**				
	**DNA extraction protocol**				Total*
	**Manual**		**Kit**		
	**Freeze & Thaw**	**Phenol-chloroform**	**Qiagen**	**Bioneer**	
Cold water tap	0/11	1/11	0/11	1/11	1/11 (9.1%)
Hot water tap	0/8	0/8	0/8	2/8	2/8 (25.0%)
Showerhead	0/9	4/9	1/9	4/9	5/9 (55.5%)
Cooling tower	0/13	2/13	2/13	4/13	4/13 (30.8%)
Hot water tank	0/4	1/4	1/4	1/4	1/4 (25%)
Total*	0/45 (0%)	8/45 (17.7%)	4/45 (8.9%)	12/45 (26.6%)	13/45 (28.9%)

*Considering overlap.

Various studies have reported different methods for DNA extraction. Hsu et al., extracted DNA from *Legionella* cells by freeze & thaw [14]. Different kits have also been used for DNA release from bacterial cells. Morio and co-workers applied a DNA Mini Kit (Qiagen) for extracting DNA in water samples to identify the presence of *Legionella*[1]. In another study, Ariefjohan and co-workers extracted total DNA from human fecal specimens utilizing four different kits [15]. They found a significant disparity in DNA extraction yield and concluded that extraction kits incorporated lysing matrix and vigorous shaking produced high quality DNA [15]. Tomaso and co-workers used five commercial kits for detection of *Brucellae* in tissue specimens. They observed significant differences in DNA yield as high as two orders of magnitude for some samples [23]. Other studies have also concluded that the extraction methods play an important role with regard to performance in downstream molecular applications [24,25].

Excluding the freeze & thaw method, in the present study a meaningful relationship was found among other three methods in which DNA extraction using Bioneer kit and to a lesser degree phenol-chlorophorm methods showed higher sensitivities. No *Legionella* were detected by freeze & thaw method, though 8.9, 17.7, and 26.6 percent of samples yielded *Legionella* contamination when Qiagen kit, classic phenol-chlorophorm and Bioneer kit methods were used, respectively. Two assumptions can be made here: a) relatively low concentrations of *Legionella* were present in these samples; b) the depletion of DNA amplification capacity rooted from the composition of water samples and presence of PCR inhibitors such as calcium and magnesium ions which may interfere with DNA extraction reagents and cause inhibition. Insufficient DNA yield due to low concentrations of *Legionella* could have led to negative results in our study when the method of freeze & thaw was conducted. Moreover, DNA yield might be out of the range of conventional PCR assay because of the small amount of bacteria in these samples that reflected the situation under real anthropogenic environmental conditions. This also indicates that DNA purification may be more relevant and a critical step when the amount of DNA in sample is very low [20]. Higher DNA yield indeed increases recovery of DNA from the bacterial community in a sample [22] and thus enhances chances of detecting rare species, such as *Legionella*. Comparing classic manual protocol and commercial kits for determination of agreement rate in our study, by excluding the freeze and thaw method in the further analysis of DNA extraction methods, showed that the interrelate analysis are Kappa coefficient calculated as 0.619 with *P* < 0.001 (Table 3). This measure of agreement is statistically significant. Most statisticians prefer Kappa values to be at least 0.6 and most often higher than 0.7 before claiming a good level of agreement [26]. Similarly, comparing Bioneer kit to the classic phenol-chlorophorm manual method illustrated the same good agreement (Kappa = 0.619); however, a moderate agreement was observed comparing phenol-chlorophorm method with Qiagen kit (Kappa = 0.433). Comparison of two commercial kits for determination of agreement rate, the results of interrelate analysis indicated Kappa coefficient calculated as 0.423 with *P* < 0.001 (Table 3). This measure of agreement inferred a statistically moderate agreement. As a rule of thumb, values of Kappa between 0.41-0.6 are considered moderate agreement. It should be also noted that a reference test is generally used to determine the sensitivity and specificity of a test or an instrument such as a kit against another one, which was not the goal of present study.

**Table 3:** **Comparison of *Legionella *** **detection results among studied protocols: Manual protocol (Phenol-chloroform) and Kit protocol (Qiagen and Bioneer)**

**Manual protocol**			**Kit**			**Kit protocol**	
		**Qiagen**		**Bioneer**			
		**No**	**Yes**	**No**	**Yes**	**No**	**Yes**
**Phenol- chloroform**	No	38	1	33	6	32	4
	Yes	4	2	6	0	7	2
Total		42	3	39	6		

In general, DNA extraction using columned Bioneer kit followed by phenol-chloroform found to be the most appropriate methods in this study as compared to others. Considering these findings, the use of a reliable, useful and adapted DNA extraction protocol in such samples is therefore of high importance.

### Legionella prevalence and species identification

**Figure 1:**
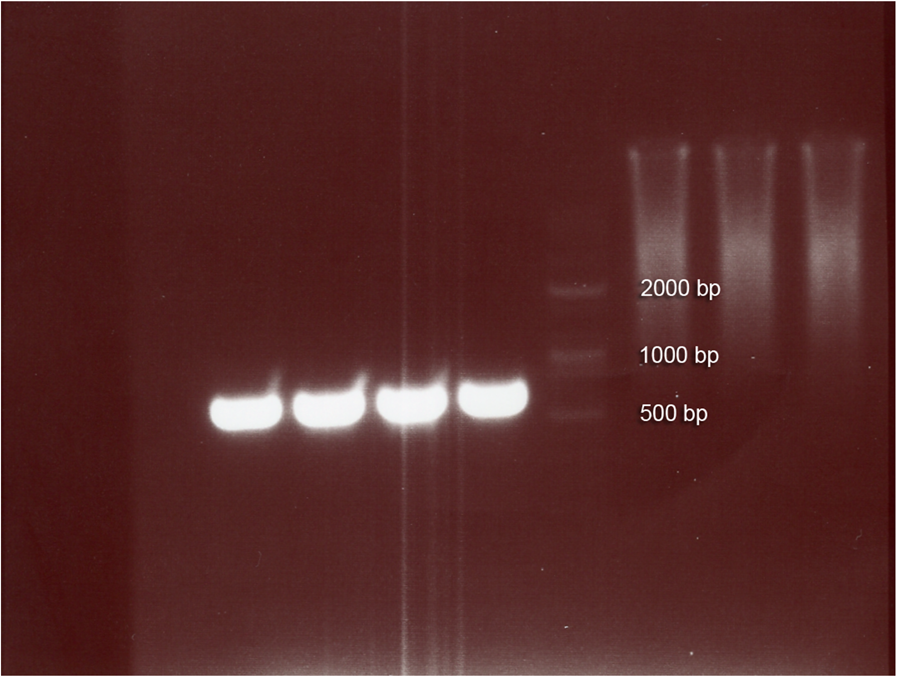
**Agarose gel electrophoresis of *Legionella*** **s' amplified DNA extracted from different hospital water sources.**

Identification of *Legionella* specie was based on the presence of an amplified product of 654 bp (Figure 1). Analysis of PCR results revealed a great diversity with regard to the sources from which samples were taken. Table 2 also demonstrates the results of *Legionella* monitoring in the seven hospitals by the source. In general, showerheads were the most contaminated source with 58.3 percent positive samples. Similarly, the samples from cooling waters, hot water taps, and hot water tanks yielded fairly comparable results (30.8%, 25%, and 25%, respectively). However, this was not the case with the cold-water tap samples and only 9% were positive for *Legionella*. For medical units with a high risk of legionellosis five positive samples in all were detected [Pediatrics stem cell transplantation (0), Bone marrow transplantation (0), Cancer Dep. (1), Maternity (1), Cardiac surgery (0), ENT (2), and Infant (1) wards].

Two positive samples were identified for species by DNA sequencing. DNA for sequencing was prepared by the 16S rRNA. The PCR products of two *Legionella* isolates were sequenced at MWG (Germany), DNA sequence was used to search the Gene Bank database, and the database entry with the highest percentage similarity was taken to identify the species. Nucleotide sequences data have been submitted to the Gene Bank database with accession Nos. FJ480932 and FJ480933.

Detection of *Legionella* in aquatic environments has been demonstrated in other researches [14,27,28]. Although PCR inhibitor may interfere with the results obtained by PCR, it has shown higher sensitivity than culture as demonstrated by Lye et al., and Morio et al., [1,29]. This may be derived from the relatively low concentrations of *Legionella* and supported by the fact that *Legionella* bacteria are commonly present in aquatic environments in the viable but non-culturable status which cannot be detected by culture [30,31]. In our study, although no statistical difference among *Legionella* positive rates in various sources was found (*P* > 0.05), the positive rate itself showed the severity of contamination. Therefore, even though the results obtained by PCR are not a valid determinant of *Legionella* viability in the environmental samples, it should be seriously considered as a potential public health threat. Owing to the lack of epidemiological and ecological studies, no *Legionella* outbreaks have been reported in environmental water samples in Tehran or other cities of Iran till now. However, considering patients’ complaint about their acquired pulmonary diseases at hospitals, the results of this study showed that sporadic or even a fairly high incidence of *Legionella* might have occurred but neglectfully distinguished as other pulmonary diseases.

## Conclusion

Considering the important role of DNA extraction methods with regard to performance in downstream molecular applications and the usual low amount of bacteria in environmental water samples such as hospital water, the use of optimised methods in detection of *Legionella* by PCR assays is critical. Molecular techniques based on PCR assay offer a rapid, practical, cost-effective and sensitive alternative for detection of *Legionella*. Although the concentration of *Legionellae* in the sampled hospital water systems was not determined, given the high positive rate of *Legionella* colonization, hospital-acquired legionellosis might be under diagnosed in Tehran. It calls for urgent control measures to minimize the transmission rate of *Legionella* from the source to the host and to prevent an outbreak.

## Competing interests

The authors declare that they have no competing interests.

## Authors’ contributions

MR, HH and AM participated in design of the study and performed experimental and molecular genetic studies. MH participated in statistical analysis. MR and MJ drafted the manuscript. All authors read and approved the final manuscript.
